# The Abortive and Curative Treatment of Gonorrhœa by the Nitrate of Silver, with Cases

**Published:** 1845-02

**Authors:** H. F. Campbell

**Affiliations:** Demonstrator in the Medical College of Georgia


### SELECTIONS

FROM

### AMERICAN AND FOREIGN JOURNALS.

---

*The Abortive and Curative Treatment of Gonorrhœa by the Nitrate of Silver, with cases; by H. F. Campbell, M.D., Demonstrator in the Medical College of Georgia.*—In the October number of the Medico-Chirurgical Review, for 1843, and also in a late number of the American Journal of the Medical Sciences, is an article on the abortive treatment of gonorrhœa, by lunar caustic. In the two communications, the reporters have applied it differently—Mr. Childs recommending its application in substance, while Mr. Debeney prefers it injected in a solution of strength, from viii. to xv. grs. to the ounce of water. Having, in the treatment of gonorrhœa, used nitrate of silver after both these modes of application, my experience goes to corroborate their testimony as to its efficacy under either form, though of the two I prefer its application by injection.

I have found that the strength of the injection prescribed by M. Debeney is not generally sufficient to relieve by the first or second application, and that it was necessary, in most cases, to increase it to from xx. to xxx. grs. to the ounce of water, and I have applied it in even a more concentrated solution. This I find to be a good modification of the two plans, inasmuch as that, while we secure its application minutely to the whole extent of the diseased surface, we, by this increase in the strength of the injection, provide a sufficiency of the agent to produce more effectual cauterization. I have had but one case, as will be hereafter seen, wherein the application has been followed by a high degree of inflammation, and in that one I have reason to suppose it to have commenced before this means of treatment had been resorted to. Generally, I have found the patient recovering speedily from both the disease and the effects of the cautery.

Case 1. A carpenter, aged 30 years, general health good; was exposed to the disease, and on the third day experienced pain in urinating, and a continued burning for some time after. He was relieved by one injection of lunar caustic, xxx. grs. to the ounce of water.

Case 2. A young man aged 17. The discharge in this case had began to show itself. Treated by one injection of lunar caustic, xl. grs. to the ounce of water; there was a discharge of blood afterwards in small quantity, but in a few days he was entirely well.

Case 3. A recent case wherein the patient complained of tenderness of the chord before treatment. One injection was made of nearly xxx. grs. to the ounce of water. The application was followed by pain in the testicle and orchitis. The patient was from that time treated by another physician; of the result I have not as yet been advised. This is the only case wherein the nitrate of silver, applied by myself in this disease, was followed by unpleasant symptoms.

Case 4. A negro man, a tailor, aged 26. A recent attack, relieved by two injections of lunar caustic, xv. grs. to the ounce of water.

Case 5. A mulatto boy aged 20 years; of a strumous diathesis; first treated by another physician, and afterwards by myself, unsuccessfully, with various balsamic mixtures and astringent injections, was cured by one injection of the nitrate of silver, xxv. grs. to the ounce of water. In this case a whitish membranous eschar was voided while urinating a few days after the application.

Case 6. A negro man aged about 23 years; a boat hand, had had the disease about ten days. One injection was applied of from xx. to xxx. grs. to the ounce of water. The balsamic treatment, before used without making any impression on the disease, was continued; the patient was well in less than a week's time.

Case 7. A white man aged 30, a team-driver, a recent case, cured by one injection, xx. grs. to the ounce of water.

Case 8. A male aged 33 years, a recent attack; discharge abundant, commenced the day before, no pain. I applied one injection of lunar caustic, xl. grs. to the ounce of water. That day there was an increase in the discharge and much pain in urinating, with some blood at that time. On the second day the discharge ceased altogether, though the pain and bloody urine continued for some days. In this case I combined, during the treatment, a few doses of balsamic emulsion each day.

Case 9. A male aged 20 years, treated on the third day after the discharge had commenced with one injection of lunar caustic, xxx. grs. to the ounce of water, together with balsamic emulsion. On the fourth day the patient was entirely cured.

Case 10. Prof. P. F. Eve here kindly furnishes me with a case wherein the balsamic and astringent treatment had proved entirely inert, though persevered in for some weeks, till combined with injections of the nitrate of silver of from viii. to xv. grs. to the ounce of water.

Of the application of the nitrate of silver in substance, I can adduce but three cases; one of which occurred in the practice of Dr. Edward A. Eve, near this city, the other two came under my own observation.

Case 11. A young man aged 26 years, sanguine temperament, treated unsuccessfully with balsamic emulsion and injections of sulphate of zinc, and sulp. of morphine. Dr. E. applied the nitrate of silver in substance, by paring the end of a cylinder, and introducing it for about a half inch or more, within the orifice of the urethra. This was done repeatedly, and the patient was relieved in a short time after this plan of treatment was adopted.

Case 12. A male aged 30 years, a mechanic, bilious temperament, general health good. In this case the gonorrhœa was complicated by a stricture, the result of previous disease, at the distance of about an inch from the orifice of the urethra; it was at a point somewhat beyond this, that the urethritis appeared to obtain, the patient experiencing pain at that place, during manipulation, and the matter confined behind the stricture, on pressure, would appear at the orifice. After dilating the stricture by bougies, I freely applied the nitrate of silver in substance, by means of a style and canula somewhat similar to those of Mr. Childs. The pain was not felt after the burning of the caustic had subsided, the discharge ceased, and in a few days the patient was well.

In the female, I have found the application of the nitrate of silver in substance, preferable to the form of injection; it is more practicable, the locality of the inflammation not being invariable. Of this class is the following:

Case 13. A white woman aged 26 years; general health good, habits regular. Treated for some weeks with the usual balsamic remedies, using at the same time very strong astringent injections of sulph. cup. and sulph. zinc. aa viii. grs.

and sulph. morphine 1 gr. to the ounce of water, applied once a day. Finding this treatment unsuccessful, I applied with a speculum uteri, the nitrate of silver, by means of a port-caustic, freely to the orifice of the urethra, mouth of the womb, and interruptedly to the sides of the vagina. The application was made but once; it caused much pain and there started a few drops of blood from the posterior lip of the os tincæ, which seemed very much congested. In this case I continued the balsamic mixture together with the above injection, diluted. The discharge, though undiminished before the cauterization, soon entirely ceased, and she was well on the fourth day.

From the consideration of the thirteen cases given above, together with the well attested experience of Mr. Childs and M. Debeney, I think we may safely conclude with regard to this mode of treatment: Firstly, that the treatment of gonorrhœa with lunar caustic, as proposed by them, is preferable to any other mode of treatment. Secondly, that the form of injection is preferable to its application in substance. Thirdly, that in cases not relieved by the injection as prescribed by M. Debeney, of strength from viii. to xv. grs. to the ounce of water, it being perfectly safe, it is advisable to increase it, even far beyond that strength. And further, that in those cases, wherein of itself it does not wholly relieve, we should by no means reject it, but continue its use as a very valuable adjunct to any other plan of treatment we can adopt.

*Southern Medical and Surgical Journal.*

